# Lower limb biomechanical characteristics of patients with neuropathic diabetic foot ulcers: the diabetes foot ulcer study protocol

**DOI:** 10.1186/s12902-015-0057-7

**Published:** 2015-10-23

**Authors:** Malindu Eranga Fernando, Robert George Crowther, Margaret Cunningham, Peter Anthony Lazzarini, Kunwarjit Singh Sangla, Jonathan Golledge

**Affiliations:** Vascular Biology Unit, Queensland Research Centre for Peripheral Vascular Disease, College of Medicine and Dentistry, James Cook University, Townsville, QLD 4811 Australia; Movement analysis Laboratory, Discipline of Sports and Exercise Science, James Cook University, Townsville, Australia; Sport and Exercise, School of Health and Wellbeing, University of Southern Queensland, Brisbane, Australia; Department of Internal Medicine, The Townsville Hospital, Townsville, Australia; Allied Health Research Collaborative, Metro North Hospital & Health Service, Queensland Health, Brisbane, Australia; School of Clinical Sciences, Queensland University of Technology, Brisbane, Australia; Department of Vascular and Endovascular Surgery, The Townsville Hospital, Townsville, Australia; Podiatry Service, Kirwan Community Health Campus, Townsville, QLD Australia; Department of Health Sciences, University of Stirling, Stirling, Scotland UK

**Keywords:** Diabetic neuropathies, Diabetic foot, Foot ulcer, Longitudinal studies, Gait, Protocols, Plantar pressure

## Abstract

**Background:**

Foot ulceration is the main precursor to lower limb amputation in patients with type 2 diabetes worldwide. Biomechanical factors have been implicated in the development of foot ulceration; however the association of these factors to ulcer healing remains less clear. It may be hypothesised that abnormalities in temporal spatial parameters (stride to stride measurements), kinematics (joint movements), kinetics (forces on the lower limb) and plantar pressures (pressure placed on the foot during walking) contribute to foot ulcer healing. The primary aim of this study is to establish the biomechanical characteristics (temporal spatial parameters, kinematics, kinetics and plantar pressures) of patients with plantar neuropathic foot ulcers compared to controls without a history of foot ulcers. The secondary aim is to assess the same biomechanical characteristics in patients with foot ulcers and controls over-time to assess whether these characteristics remain the same or change throughout ulcer healing.

**Methods/Design:**

The design is a case–control study nested in a six-month longitudinal study. Cases will be participants with active plantar neuropathic foot ulcers (DFU group). Controls will consist of patients with type 2 diabetes (DMC group) and healthy participants (HC group) with no history of foot ulceration. Standardised gait and plantar pressure protocols will be used to collect biomechanical data at baseline, three and six months. Descriptive variables and primary and secondary outcome variables will be compared between the three groups at baseline and follow-up.

**Discussion:**

It is anticipated that the findings from this longitudinal study will provide important information regarding the biomechanical characteristic of type 2 diabetes patients with neuropathic foot ulcers. We hypothesise that people with foot ulcers will demonstrate a significantly compromised gait pattern (reduced temporal spatial parameters, kinematics and kinetics) at base line and then throughout the follow-up period compared to controls. The study may provide evidence for the design of gait-retraining, neuro-muscular conditioning and other approaches to off-load the limbs of those with foot ulcers in order to reduce the mechanical loading on the foot during gait and promote ulcer healing.

**Electronic supplementary material:**

The online version of this article (doi:10.1186/s12902-015-0057-7) contains supplementary material, which is available to authorized users.

## Background

Foot ulceration is the main precursor to lower limb amputation in patients with diabetes worldwide [1]. Estimates of the prevalence of diabetic foot ulcers (DFUs) in the United States range between 4 and 12 % [1]. The annual and lifetime incidence of DFUs has been estimated as approximately 4 and 25 %, respectively [1]. In Queensland, Australia, 24,917 hospital admissions were used for the principal management of a diabetes related foot complication between 2005 and 2010 resulting in the use of 260,085 hospital occupied bed days [2]. Treatment of DFUs account for significant health care costs in Australia and foot complications are the second leading cause of diabetes related mortality, second only to cardiovascular disease [3]. Most foot ulcers are triggered by diabetes associated peripheral neuropathy (DPN) [4, 5]. DPN which is believed to be a consequence of uncontrolled hyperglycaemia, prompts gradual loss of sensory, motor and autonomic function of the lower limb which leads to foot deformities, abnormal gait and higher plantar pressures which promote skin trauma and subsequent DFUs [4, 6–8].

Although biomechanical studies have helped to identify potential triggers of ulceration, whether such triggers change and by how much when DFUs are healing remains largely unknown [9, 10]. It is unknown whether patients with DPN are capable of adjusting their gait and plantar pressure (i.e. reducing pressure) to account for plantar wounds, as is the case for healthy controls with normal peripheral sensation [9]. It can be hypothesised that due to DPN, patients with active plantar DFUs would continue to demonstrate similar abnormal lower limb biomechanical characteristics as displayed prior to the DFU formation [10]. Biomechanical factors associated with DFUs include reduced temporal spatial parameters (TSPs), such as speed of walking and stride length; restricted kinematics (movement patterns); delayed muscle activations; and altered forces (kinetics),which may contribute to elevated plantar pressures during gait [9]. It may be possible that the patients who achieve successful DFU healing are able to compensate for the DFU through changes to these biomechanical factors, irrespective of external devices used to offload ulcers. Findings from a recent meta-analysis suggested that patients with active DFUs have reduced plantar pressure compared to those with DPN without ulcers, contrary to what was previously thought [9]. We proposed that a ‘guarded gait strategy’ may be adopted by patients with active DFUs to potentially achieve successful DFU healing leading to reduced plantar pressure [9]. To our knowledge, this concept has not previously been investigated. It is likely that exploring the gait and plantar pressure of patients with DFUs during barefoot walking (without the influence of off-loading devices or shoes) over-time will advance knowledge of how gait changes during DFU healing.

Detailed information about the biomechanical factors in patients with active DFUs may provide evidence for the design of gait-retraining and neuro-muscular conditioning and more efficient offloading methods to reduce the mechanical loading on the foot during gait [10–12]. Findings may also provide a means to select the most appropriate patients for different off-loading methods to improve DFU healing. The primary aim of this study is to establish the lower limb biomechanical characteristics (TSPs, kinematics, kinetics, muscle activations and plantar pressures) of patients with plantar neuropathic DFUs. The secondary aim is to assess the changes in these characteristics at 3 months and 6 months follow-up. This protocol details the proposed methodology for this planned study.

## Methods/Design

### Study design and participants

**Fig. 1:**
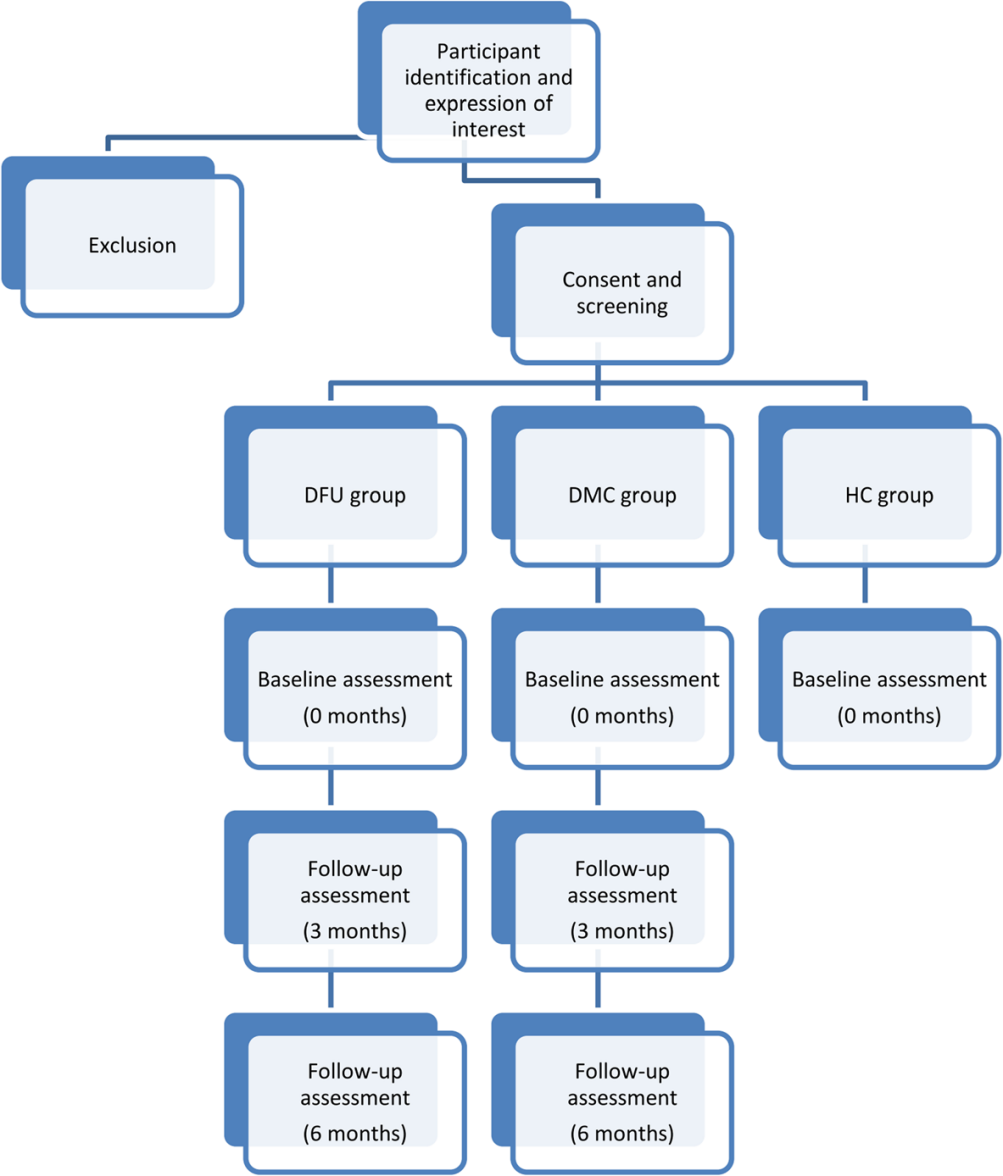
Outline of the study. Legend: DFU group = type 2 diabetes mellitus patients with a unilateral plantar foot ulcer, DMC group = patients with type 2 diabetes mellitus without a history of foot ulcers, HC group = healthy controls without type 2 diabetes mellitus or a history of foot ulcers

A case–control study nested in a six month longitudinal study was planned. The study will be performed in Townsville, a regional town in north Queensland, Australia with an approximate population of 175,000 and an estimated type 2 diabetes prevalence of 4.4 % [13]. Type 2 diabetes patients with active unilateral plantar DFUs (DFU group), type 2 diabetes control patients without a history of DFUs (DMC group) and healthy controls without diabetes or DFUs (HC group) will be recruited for this study. We will attempt to match the sex of both control groups with that of cases and match age within a range of 5 years. A 1:4:2 ratio of cases to controls [DFU: DMC: HC] is planned due to the expected difficulty in recruiting DFU patients in comparison to controls. Figure 1 outlines the proposed study protocol. All assessments will be carried out by the principal investigator (MEF), who is a trained podiatrist undertaking a PhD.

### Definitions of outcome measures and families of biomechanical outcomes

The primary and secondary outcome measures of the case control and longitudinal studies include several biomechanical variables. These variables will be grouped into gait and plantar pressure outcomes and will contribute to four main families of hypotheses. The four families of biomechanical hypotheses are 1) abnormal TSPs, such as speed of walking and stride length; 2) restricted kinematics (movement patterns); 3) altered kinetics, (altered forces) and 4) elevated plantar pressures on the ulcerated and non-ulcerated foot [9]. The kinematics can be further divided into three families; 5) sagittal plane kinematics, 6) frontal plane kinematics and 7) transverse plane kinematics. Therefore there are seven families of hypotheses in total. The main hypothesis is that patients with active plantar DFUs have altered lower limb biomechanical characteristics compared to healthy and diabetes controls. The follow-up hypothesis is that the biomechanical characteristics of patients with DFUs will not significantly change over six months follow-up compared to the DMC group.

Primary outcome measures relating to plantar pressure will include the:

- Mean peak pressure: The mean peak pressure (average peak pressure), in N/cm^2^ recorded within the plantar aspect of foot at ten anatomical sites;
- Maximum peak pressure: The maximum peak pressure in N/cm^2^ recorded within the plantar aspect of foot at ten anatomical sites;
- Total contact area of the foot: The area of the plantar aspect of the foot in contact with the pressure platform in cm^2^ at ten anatomical sites;
- Estimated vertical ground reaction force: The contact area multiplied by the mean peak plantar pressure in Newtons (N) at ten anatomical sites.

Primary outcome measures relating to gait will include:

- TSPs (walking speed, cadence, stride time, step time, opposite foot off time, opposite foot contact, foot off time, single support time, double support time, stride length and step length);
- Kinematic variables (angular joint movement characteristics of the pelvis, hip, knee and ankle bilaterally in the sagittal, transverse and frontal planes during the gait cycle);
- Kinetic variables (forces): Reported as the vertical, medial-lateral and anterior-posterior components of ground reaction force during the gait cycle in Newtons (N);

Secondary outcome measures for gait will include:

- Muscle voltages for six lower limb muscles including the vastus medialis, peroneus longus, tibialis anterior, semitendinosus and medial and lateral gastrocnemius during the gait cycle;
- Joint accelerations and velocities, in the sagittal, transverse and frontal planes during the gait cycle, reported as degrees per second (°/s) and degrees per second squared (°/s^2^).
- Joint powers during gait: Reported as the estimated power generated by the muscles acting at the hip, knee and ankle during gait, in Watts (W);
- Joint moments generated at the hip, knee and ankle bilaterally in the sagittal, transverse and frontal planes during the gait cycle, reported in Newton meters (Nm).

### Sample size calculations

We plan to examine multiple hypotheses in this study. Sample sizes were calculated to test the hypothesis that forefoot plantar pressures were different in patients with active DFUs compared with controls. This hypothesis was chosen for the sample size calculation since previous studies suggested this measure had large between patient variability and therefore was likely to require the largest sample size [14]. The effect size for this calculation was based on previous research where higher plantar pressures were documented in patients with DPN without a DFU history compared to healthy controls without either DPN or DFUs [15]. The sample size was based on an expected mean (standard deviation) forefoot plantar pressure of 3.2 (1.0) N/cm^2^; 3.9 (1.0) N/cm^2^; and 4.7 (2.0) N/cm^2^ for the HC, DMC and DFU groups, respectively. It was estimated that those with DFUs would have at least a 20 % higher plantar pressure than the control groups due to the observed difference in means of those with DPN and healthy controls [15]. We also accounted for the potential difficulty in recruiting active DFU cases in our sample size calculations by inflating the control to case ratio. Therefore, a ratio of 1 DFU; 4 DMC; 2 HC was used. We estimated that 28, 112 and 56 participants were required in the DFU, the DMC and the HC groups, respectively, using a one-way analysis of variance (ANOVA) with 80 % power and an overall significance of 0.05 (maximum of 8 planned hypotheses to be tested). The planned hypotheses to be tested are:

- The DFU group will demonstrate significantly poorer TSPs compared to controls at baseline.
- The DFU group will demonstrate significantly restricted kinematics at the ankle, knee, hip and pelvis compared to controls at baseline.
- The DFU group will demonstrate significantly altered kinetics (anterior posterior, medial-lateral and vertical ground reaction forces) compared to controls at baseline
- The DFU group will demonstrate significantly different plantar pressure. characteristics (mean peak, maximum peak, contact area and localised vertical ground reaction forces) compared to controls at baseline.
- The TSPs in the DFU group will remain poorer at 3 and 6 months follow-up compared to the DMC group.
- The kinematics of the DFU group will remain different at 3 and 6 months follow-up compared to the DMC group.
- The kinetics of the DFU group will remain different at 3 and 6 months follow-up compared to the DMC group.
- The plantar pressure characteristics of the DFU group will remain different at 3 and 6 months follow-up compared to the DMC group.

Due to the large number of hypotheses, we planned to correct for multiple testing which is described in detail in the statistical analyses section. We inflated the sample sizes to account for a proposed 10 % drop-out rate during the six month follow-up and therefore 31, 123 and 58 participants were required in the DFU, the DMC and the HC groups, respectively. An interim analysis of the study is planned after recruiting 50 % of the estimated number of participants for each group. This is to re-assess the proposed sample sizes since the initial effect size was based on patients that had DPN but no DFUs. A post-hoc power test will be performed to re-assess actual statistical power when recruitment is concluded. This test will be focused on plantar pressure values measured in the forefoot. All sample sizes were performed using the G*Power statistical software (G* Power version 3.1, Faul, Erdfelder, Lang, & Buchner, Germany) [16].

### Sample selection and setting

The DFU and DMC patients will be recruited from inpatient wards, outpatient clinics and community health clinics within the Townsville Hospital and Health Service District, Queensland, Australia. The HC group will be recruited through community advertising and from staff at the university where the study will take place. The study is approved by The Townsville Hospital and Health Service District and the James Cook University human research ethics committees (approval numbers HREC/12/QTHS/77 and H4693). Written informed consent will be obtained from all participants at the time of screening for participation in the study and for publication of de-identified data.

### Inclusion criteria

Inclusion criteria for the cases (DFU group) include: males and females above the age of 18 years; a diagnosis of type 2 diabetes (confirmed on review of the patient’s hospital or General Practitioner record) and a single active unilateral plantar DFU of >3 months duration which is not completely epithelialized. The DMC group will comprise males and females above the age of 18 years, with a diagnosis of type 2 diabetes (similarly defined as for the DFU group) and no history of plantar DFUs. The HC group will comprise participants who do not have either a diagnosis of type 2 diabetes or any foot ulcer history as assessed on interview and examination.

### Exclusion criteria

All participants will be required to be able to ambulate without any assistance or mobility aids. The exclusion criteria were designed to exclude participants with problems impacting on mobility which would likely mask the impact of plantar DFUs on gait. Exclusion criteria for recruitment to all three groups are: (1) chronic or acute orthopaedic, musculoskeletal, vestibular, visual or neurological problems affecting mobility (other than DPN) at the time of screening; (2) any history of orthopaedic surgical intervention of the lower limb (including knee arthroplasty, ankle reconstruction, bunionectomy and any other orthopaedic surgical intervention of the hip, knee or foot (besides history of surgical debridement of tissue in the DFU group); (3) presence of any form of diabetes other than type 2 diabetes; (4) planned vascular reconstruction; (5) pregnancy; and (6) systemic disease affecting mobility or leading to chronic inflammation of any lower limb joint.

All participants will need to demonstrate visual acuity adequate for walking. This will be assessed by asking the participant whether they had visited an optometrist in the last twelve months and whether they had difficulty walking due to visual impairment. If they have not visited an optometrist, participants would be required to do this before being considered for the study. Visual acuity will then again be checked prior to group allocation. We do not plan on excluding participants with diabetic retinopathy as this is an associated microvascular condition of diabetes, provided that participants do not disclose limitations in their vision that affected their walking ability. All participants unable to fulfil these criteria will be excluded from the study. Peripheral artery disease will be excluded as a major contributing cause of foot ulceration by a number of methods. Firstly the DFU patients will be reviewed by a board accredited vascular specialist (JG) who will take a relevant history and perform clinical examinations to exclude significant peripheral artery disease. DFU patients will need to demonstrate an absence of symptoms of intermittent claudication [17] and an ankle-brachial pressure index (ABPI) of >0.8 bilaterally, for inclusion. Participants enrolled in the control groups will also need to demonstrate an absence of symptoms of intermittent claudication [17] and an ABPI of >0.8 bilaterally. Any participants who demonstrate ABPIs of >1.3 will also be referred to the vascular specialist for review of inclusion [18]. Participants will not be excluded on grounds of a history of peripheral vascular reconstruction provided they demonstrate adequate peripheral circulation at the time of screening as based on the criteria listed above.

### Baseline measurements

#### Clinical history and demographic assessment

A previously developed and detailed case report form (see Additional file 1: Supplementary file S1) will be used for collecting data. A medical history will be obtained from participants via a structured interview using the case report form at baseline and follow-up visits. The case report form will be used to collect information on: Demographic details; history of significant medical conditions and treatments; medication history; ethnicity, particularly in relation to indigenous status [19]; diabetes history, such as duration of diabetes and family history of diabetes [20, 21]; smoking history [22]; walking and exercise habits [23]; and footwear use [24]. Participants will be interviewed by a single researcher (MEF). The hospital or clinical charts of patients with type 2 diabetes will also be reviewed. Medical conditions recorded will include: Hypertension, defined as history of diagnosis or treatment with antihypertensive medication; dyslipidaemia, based on history or treatment with hypolipidemic agents; stroke, defined as a history of an ischemic or haemorrhagic stroke; coronary heart disease, defined as history of stable angina or unstable angina or myocardial infarction; chronic heart failure, based on clinical history of congestive heart failure; chronic pulmonary disease, based on clinical history and/ or treatment; chronic liver disease, based on clinical record; chronic kidney disease, defined as an estimated glomerular filtration rate (eGFR) of <60 ml/min/1.73 m^2^ for 3 months recorded in the medical records. These risk factors are either known to influence foot ulcer development or healing [25], are considered as complications or conditions associated with type 2 diabetes [21, 26, 27], or are related to general health and function [28].

### Blood tests

Information will also be collected regarding blood markers that are obtained as part of clinical care of participants with type 2 diabetes. Blood markers to be recorded include HbA1c, circulating lipids and eGFR measured in a local pathology department. Ion-exchange high-performance liquid chromatography (HPLC) will be used to estimate HbA1c as a measure of long-term glycaemic control with the RIANT II TURBO Link System (Bio-Rad Laboratories, USA) [29]. Serum lipids will be measured by separating serum from the whole blood and performing analysis for total cholesterol, triglycerides, low-density lipoprotein (LDL), and high density lipoprotein (HDL) using automated assays on an Abbott Architect ci8200 machine (Abbott Park, IL, USA) [30]. eGFR will be calculated using the Chronic Kidney Disease-Epidemiology Collaboration group (CKD-EPI) formula, which is validated for Australian populations [31, 32].

### Anthropometric assessments

Physical examination will involve the assessment of height, weight, body mass index (BMI), body fat percentage and waist and hip circumference. All clinical and anthropometric measures (described below) will be carried out three times and averaged. Participant’s height will be measured using a wall mounted telescopic metal stadiometer (Seca model 220, Seca Scales, Hamburg, Germany). Percentage body fat (% body fat) will be estimated using bioelectrical impedance scales (TANITA TBF 521, TANITA Corporation, Arlington heights, Illinois, USA) which also measures weight (Kg) [33]. BMI will be calculated by dividing the participant’s body mass (Kg) by the square of the participant’s height (m). A standardised metal measuring tape (KDSF10-02, KDS Corporation, Osaka, Japan) will be used to assess hip and waist circumference while the participant is in a relaxed stance positon with feet together and arms freely hanging to the side. The measurement will be performed at the end of exhalation [34]. Waist circumference will be measured at the natural waist i.e. in between the lowest rib and the top of the iliac crest at the narrowest point of the lower abdomen [34]. Hip circumference will be considered as the widest portion of the buttocks, with the tape parallel to the floor [35]. Measurements will be repeated three times and averaged [34].

### Blood pressure and peripheral arterial examination

Blood pressure (systolic and diastolic) will be measured using an electronic blood pressure machine (Connex ProBP 3400 digital blood pressure device; Welch Allyn, New York, USA) mounted on a mobile stand [36]. Systolic and diastolic measurements will be taken in both arms while the participant is sitting and relaxed, and repeated three times, two minutes apart [37]. The highest averaged value from the two arms will be used to represent participant’s blood pressure. Blood pressure measurements will be performed using an appropriately sized sphygmomanometer cuff (i.e. using a large-adult cuff size for individuals with an arm circumference of 33 cm or greater) [38].

The peripheral arterial examinations will first comprise the palpation of the dorsalis pedis and posterior tibial pulses for their presence or absence as well as strength (categorised as normal or reduced) [39]. The ABPI will be measured in each lower limb using previously validated methods [40]. The ABPI will be performed with the participant lying on a plinth and after being rested for ten minutes. A 5 MHz Doppler probe (MD 6, Hokanson, Bellevue, USA) angled at 45° to the direction of the blood flow will be used to assess the ABPI. The brachial artery pulse will be manually palpated and the Doppler probe will be used to detect a signal. The sphygmomanometer cuff will be inflated until the Doppler signal disappears and deflated slowly until the signal returns. The pressure at which the signal returns will be recorded as the brachial artery Doppler pressure. Three brachial artery Doppler pressure measurements will be taken in each arm using this method. The sphygmomanometer cuff will then be placed around the participant’s ankle immediately above the medial and lateral malleolus and the Doppler probe will be placed on the dorsalis pedis artery after palpation. The above mentioned process will be repeated three times to assess the dorsalis pedis artery Doppler pressure. A similar process will be carried out to assess the posterior tibial artery Doppler pressure. The highest average Doppler pressure measurement in each ankle will be divided by the highest average brachial pressure to obtain an ABPI value for each foot. We utilised the combination of ABPI and claudication symptom questionnaire to identify peripheral artery disease as previously reported [17]. This combination is thought to provide a better means of detecting peripheral artery disease, especially since falsely elevated ABPI values are common in patients with diabetes mellitus [18, 41].

### Screening for diabetic peripheral neuropathy presence and severity

Neurological examination will consist of a 10 g (size 5.07) monofilament sensation test [42], a 128 Hz tuning fork sensation perception test [43] and administration of the Michigan neuropathy screening instrument (MNSI) (Michigan Diabetes Research and Training Center, 2013) [44]. The MNSI is a tool which has been validated for assessment and quantification of the degree of peripheral sensory and motor neuropathy present in participants with diabetes [44]. This includes asymptomatic peripheral neuropathy [45]. The tool contains both a symptom and physical component [46]. A score out of 11 will be generated for the symptom related questions of the MNSI and a score out of eight will be generated for the physical assessment component.

Monofilament sensation will be assessed at ten sites (two additional sites compared to previous reports) on each foot including the plantar surfaces of the heel on the medial and lateral sides, hallux, apex of toes 2–5, metatarsal one, metatarsal five and the dorsum of the foot between the first and second metatarsals [47]. The examination will be performed with the participant lying supine in a relaxed position with their eyes closed. The participant will be given the opportunity to feel the sensation in their index finger prior to testing in the foot. The monofilament will be applied for <1 s at each site and the participant will be requested to verbally acknowledge whether they felt the sensation and to acknowledge the site of sensation [48]. A score of five or less out of eight has been reported to indicate the presence of peripheral neuropathy based on assessing eight sites [47]. We will utilise this grading system with the inclusion of two additional sites to encompass the main regions of the whole foot. Where a participant is unable to detect the monofilament at five or less sites they will be considered to have DPN [47]. Additionally a 128 Hz tuning fork will be applied to the tip of the hallux at the bony prominence of the distal phalanx on both feet to check whether vibration sensation is present [43, 49]. Patients will be asked to verbally indicate the commencement and cessation of vibration sensation upon application and dampening of vibration four times on each hallux [43]. Vibration sensation will be assessed on a scale of 0 to 8 in each leg based on the number of times vibration commencement and cessation is felt accurately [49]. The scores obtained will be reported and compared between groups.

### Physical examination of the lower extremities

The lower limb examination will encompass an assessment of the static range of movement of the ankle joints, sub-talar joints and the first metatarsophalangeal joints using previously described methods [50, 51]. A goniometer will be used while the participant is relaxed and lying on an examination plinth and while the patient’s legs are in complete extension [50, 51]. Ankle and sub-talar joint range of motion will be recorded as a categorical outcome where the movement will be classified as restricted, normal or hypermobile based on the range of movement of the joint [52]. The muscle power of the lower limbs will be assessed using the Medical Research Council (MRC) scale [53] while the participant is lying supine. The degree of movement of the foot in the sagittal, coronal and frontal planes will be documented during abduction/adduction, plantar flexion/dorsiflexion and inversion/eversion. MRC grading will be evaluated as a categorical value with a score less than five indicating a restriction in movement [53]. Other assessments will include examination of the medial longitudinal arch contour (both on stance and during sitting) [54], an examination for lesser toe deformities (claw, hammer and mallet toes) [55] and the identification of the presence and degree of hallux valgus (HAV) deformity of the first metatarsophalangeal joints [11, 56].

### Assessment and classification of plantar foot ulcers

The University of Texas Wound Classification System (UTWCS) is a validated tool for the measurement of foot ulcers [57]. The UTWCS will be used to grade the ulcer for severity based on the presence of infection, ischemia and depth. Photographs will be taken of each participant’s DFU at each visit and wound measurements will be conducted by MEF to measure depth, width and length of the ulcer in cm and the surface area will be calculated using the formula length (cm) x width (cm) = surface area (cm^2^) [58]. Other information such as the ulcer location, the estimated duration of the ulcer (in weeks; recorded at the initial date of presentation within the notes to the time of assessment), the wound exudate level, the type of exudate, the appearance of the wound bed (base of the wound) and wound edge, and the presence of sinus formation will also be collected [58, 59]. If clinical signs of infection are apparent (using Infectious Diseases Society of America Clinical Practice Guideline for the Diagnosis and Treatment of Diabetic Foot Infections [60]) then a wound swab will be performed [61, 62] to identify the presence of bacterial infection as a part of treatment of the ulcer as reported in current guidelines [62–64].

The type of offloading utilised and the type and frequency of wound dressings will also be collected [63, 65, 66]. All patients with DFUs will be given a standard single-layer generic film wound dressing which they will wear over the wound during gait examination to minimise the impact of wound dressings on the study results and to standardise the type of dressing during gait assessments. As a precautionary measure, participants with DFUs will only be requested to weight bear while gait and plantar pressure assessments are being carried out and they will be instructed to remain sitting with their feet off the ground as much as possible between assessments.

### Assessment of gait

The three dimensional movement analysis component of the study will involve attaching reflective markers to the participant’s skin over the surface of key anatomical landmarks [67]. This enables the assessment of movement characteristics such as TSP’s, joint kinematics, power and moments [68]. The movement analysis laboratory at James Cook University will be used for gait assessments. This lab is equipped with the VICON gait analysis system (VICON, Oxford, United Kingdom). The system has ten T-40 series infrared cameras positioned around a walking environment capturing data at 100 Hz within the VICON nexus movement analysis software (version 1.9.1, VICON, Oxford, United Kingdom). The force plates in the laboratory comprise of two 400 × 600 mm OR-6 AMTI force plates and two 900 × 900 mm OR-6 AMTI force plates (AMTI, Watertown, Massachusetts, USA) which are embedded on a 10 m long walking surface covered by concrete overlay. The force plates have a maximum excitation range of 10 volts with each occupying six analogue channels (<2 % channel cross talk). In addition to this, two Bortec AMT 8 Electromyography systems (Bortec Biomedical, Alberta, Canada), comprising 16 individual channels will be utilised to capture electromyographic data. The force plates and electromyographic unit are programmed to capture at a rate of 3000 Hz (3000 frames per second), for optimum capture speed whilst utilising all equipment simultaneously. All equipment are linked and synchronized with the VICON system in the laboratory. A similar system was used in a recent study investigating gait features of patients with trans-tibial amputation [69] and in another study assessing gait in patients with a history of foot ulcers [70].

A standard VICON Nexus procedure will be used during motion capture (Vicon Motion Systems, Oxford, England) [71]. Ten walking assessments while walking at a self-selected pace will be collected from each participant. These ten walking assessments will be averaged and compared across groups.

### Assessment of plantar pressure

The Footscan® pressure plate (RSScan International, Olen, Belgium) will be used for plantar pressure assessment with the associated Foot Scan® processing software. This platform is two meters in length, 0.4 m in width and contains 16,384 sensors capturing at 100 Hz. The plantar pressure platform is freestanding. The platform has been used for previous biomechanical research in participants that have diabetes [72]. Ten walking assesments will be collected from participants while walking at a self-selected pace using the three step-protocol [73]. This involves the participant being trained to approach the plantar pressure platform so as to strike their third step (i.e. contact of the initiating limb) on the pressure platform first followed by the opposite foot second [73]. The pressure measurement software permits masking of the foot to enable identification of plantar pressures at a total of 20 anatomical locations in both feet. This allows for the quantification of plantar pressure based on pre-established anatomical locations [7]. The locations include the plantar surfaces of the hallux, combined toes one to five, metatarsal one, metatarsal two, metatarsal three, metatarsal four, metatarsal five, the mid foot, the lateral rear foot and the medial rear foot. These measurements are reported by the software as the mean peak pressure (mpp), maximum sensor pressure (msp), pressure time integral (pti) and contact area (ca). A consistent measurement will be defined as a walking assessment in which all ten anatomical locations were visible with a numerical value for mpp, msp, pti and ca in both feet and where lateral or medial deviation of the foot off the pressure platform did not occur [7].

### Measurements to be repeated at the follow-up assessments

Follow-up assessments will be performed on all participants in the DFU and DMC groups at three and six months. The HC group will be only examined at baseline as it is anticipated that the gait and plantar pressure of these participants will not change during short term follow-up [74]. The case report form (see Additional file 1: Supplementary file S1) will be used for collecting data at each follow-up visit. Follow-up assessment will involve a physical examination, including assessment of height, weight, BMI, % body fat and waist and hip circumference. A questionnaire contained within the case report form will be used to assess any changes to participants’ medical history or medication. This form will also be used to document foot ulcer recurrence, new ulcer formation and ulcer healing, new treatments and foot ulcer characteristics (if applicable). Gait and plantar pressure measurements will be performed at the three and six months follow-up visits to assess any changes to the primary and secondary outcome measures. Eligibility to participate will be reassessed at each visit and any reasons for discontinuation of the study will be recorded and reported.

### Statistical analyses

The Statistical Package for the Social Sciences (SPSS) version 22.0 for Windows (SPSS Inc., Chicago, IL, USA) will be used for statistical analyses. Continuous data will be reported as mean ± standard deviation or median and interquartile range based on the distribution of data in the three groups. The Shapiro-Wilk test will be used to test for normality. All primary and secondary outcome variables will first be assessed graphically using scatter and box plots and using correlation analyses to look at between group differences in data. If the data is of normal distribution, parametric analyses will be used. Explanatory variables will be compared between the groups using one way ANOVA and between the DFU and DMC groups using student *t*-test for continuous variables. Non-parametric analyses with the Kruskal Wallis test and the Man Whitney *U* test will be used if the data is not normally distributed. Pearson’s chi-square tests or Fisher’s exact tests will be used for categorical variables. Fisher’s exact test will be used where the expected frequencies are less than or equal to five. Similarly, primary and secondary continuous data outcomes will be initially analysed using one way ANOVA or Kruskal Wallis test based on distribution of the data. Outcomes will be corrected for multiple testing where more than the planned number of hypotheses are tested and where more than the planned four families of hypotheses are tested. The Holm correction will be used for correcting p-values of unplanned tests [75]. This will correct for type 1 error. Type 2 errors are controlled by using sample size calculations. P-values from the test statistic and 95 % confidence intervals of the mean will be reported for each group for each outcome measure as a measure of significance. Effect-sizes of each significant outcome measure will be reported using the Cohen D value in reference to previously reported biomechanical effect sizes [76]. Where possible and appropriate, analyses using binary logistic regression will be performed to adjust for potential confounding including age, gender and BMI, despite matching, for all significant outcomes. Follow-up data will be analysed using either repeated measures ANOVA or a similar repeated measures analysis of variance statistical technique such as generalised linear mixed models, utilising time as a co-variate to assess changes in gait and plantar pressure variables over-time. Alpha will be set to 0.05 for all analyses.

## Discussion

We report the methodology for a longitudinal study investigating the gait and plantar pressure characteristics of patients with active DFUs. There is a paucity of studies investigating the biomechanical characteristics of patients with active neuropathic DFUs and thus a need to better document these characteristics. Altered biomechanical characteristics are likely to influence the healing of plantar DFUs. It may be possible to address altered biomechanical characteristics to reduce the mechanical loading on the ulcerated foot during gait using gait retraining techniques in addition to off-loading the foot. The design of these interventions is likely to be improved by further understanding of gait during the presence of an ulcer. We believe this study will provide important observations regarding the association between gait and foot ulcer healing.

## Additional file

Additional file 1:
**Supplementary data file S1.** PDF document containing the case report form for data collection. (PDF 1054 kb)

## Abbreviations

DPN: Diabetic peripheral neuropathy
DFUs: Diabetic foot ulcers
DMC: Diabetes mellitus control
HC: Healthy control
ASIS: Anterior superior iliac spine
ABPI: Ankle brachial pressure index
TSP: Temporal spatial parameters
mpp: Mean peak pressure
msp: Maximum sensor pressure
pti: Pressure time integral
ca: Contact area
CCC: Concordance correlation coefficient
CV: Coefficient of variation
MRC: Medical research council
HAV: Hallux valgus deformity
