# Supplementary material for: Lower limb biomechanical characteristics of patients with neuropathic diabetic foot ulcers: the diabetes foot ulcer study protocol
Source: BMC Endocr Disord. 2015 Oct 23;15:59. doi: 10.1186/s12902-015-0057-7 (PMC4619003; doi:10.1186/s12902-015-0057-7)
Supplement: Additional file 1: — Supplementary data file S1. PDF document containing the case report form for data collection. (PDF 1054 kb) [file 12902_2015_57_MOESM1_ESM.pdf]

Supplement 1: Case Report Form

**Protocol Title:**	**Full Title:**<br>**Lower limb biomechanical characteristics of patients with neuropathic foot ulcers (DFU study)**<br><br>**Short title:**<br>**Diabetes and Foot Ulceration Study**
**Participant Initials:**	____ _____
**Unique identification number**	D F U ___ ___ ___
**Date of Birth:**	___ ___/___ ___ ___/___ ___ ___ ___
**Site:**	The Townsville Hospital- Diabetes Clinic, Department of Endocrinology<br>&<br>James Cook University Movement Analysis Laboratory
**Scheduled date of first Gait analysis investigation**	___ ___/___ ___ ___/___ ___ ___ ___
**Participant Alerts: (Allergies or specific requirements)**	
**Date Completed:**	___ ___/___ ___ ___/___ ___ ___ ___

Queensland Government
Queensland Health

JAMES COOK UNIVERSITY
AUSTRALIA

Case Report Form (CRF) Completion Instructions

Please ensure the headers are completed on each page

- Participant Initials should be recorded as a three-letter sequence of the participant's initials (First, Middle, Last). If the participant does not have a middle name use a dash (-)
- Patient identification-number for the study.

Complete all pages in a medium to heavy point black ink pen ONLY

Ensure all figures are written inside the designated space, try not to touch the boundaries of this space to maximise accuracy

All text and explanatory comments should be brief and written within the space provided

To answer multiple choice questions place a cross (X) inside the designated space

DO NOT use liquid paper or permanently remove or cover an error. To make a correction, drawn a single line through the original value and write the new value as close as possible to the original space. Initial and date the correction

Do not fold the forms.

If there are any questions please contact the principal study investigator:

Mr. Malindu (Mal) Fernando
Podiatrist and Cohort Doctoral Candidate
James Cook University, Faculty of Medicine and Molecular Sciences.
Telephone: +61 7 47813144
Fax: + 61 7 47813179
Email- malindu.fernando@my.jcu.edu.au

Visit 1, Part 1- Site The Townsville Hospital.

Eligibility Criteria

Inclusion Criteria

*All criterion listed below must be answered **YES** for the participant to be considered eligible*

1	Has the participant been clinically diagnosed with Type II diabetes mellitus or receiving medication for this?	☐ Yes ☐ No
2	Is the participant able to walk unassisted, utilising two feet?	☐ Yes ☐ No
3	Has the participant agreed to comply with all study procedures and instructions?	☐ Yes ☐ No

Exclusion Criteria

*All criterion listed below must be answered **NO** for the participant to be considered eligible*

1	Does the participant have type I diabetes	☐ Yes ☐ No
2	Is the participant an amputee, either BKA or have any foot amputation?	☐ Yes ☐ No
3	Does the participant have a known vascular reconstruction or orthopaedic surgical procedure scheduled or planned within the next 12 months?	☐ Yes ☐ No
4	Has the participant previously had foot and ankle surgery?	☐ Yes ☐ No
5	Does the participant have a bleeding disorder?	☐ Yes ☐ No
6	Is the participant under 18 years of age?	☐ Yes ☐ No
7	Does the participant require ambulatory assistance?	☐ Yes ☐ No
8	Is the participant pregnant?	☐ Yes ☐ No

Note- If the patient has a present plantar foot ulcer, allocate patient to case group and if not, allocate patient to control group.

Is the participant a healthy control? **Yes** **No**

Diabetes and Foot ulceration Study		
Unique Identification Number:	UR:	Name:
DOB:	Initials:	

Visit 1

Informed Consent

PICF version	Patient Has consented to sections<br>A B C
Date on which Informed Consent was obtained ☐☐ /☐☐☐/☐☐☐☐<br>(dd/mm/yyyy)	

Demographics

Date of birth ☐☐/☐☐☐/☐☐☐☐ (dd/mm/yyyy)	
Gender	☐ Male ☐ Female
**Ethnicity**<br>Caucasian<br>Asian<br>African<br>Aboriginal/ Torres strait islander<br>Other ________________________	
Aboriginal or Torres strait Islander?	Yes No

Smoking status

Never smoked ☐	
Ex-smoker (has not smoked in the last month)* ☐	Current smoker* ☐
*If an ex- or current smoker please answer the following	
Number of years smoking ☐☐	
Average number of cigarettes per day ☐☐	

Medical History

Please note that the definition of these conditions is based on a history of diagnosis or treatment

Hypertension	☐ Yes ☐ No
Dyslipidemia	☐ Yes ☐ No
Stroke/ TIA – Date _______________________	☐ Yes ☐ No
Peripheral Arterial Disease	☐ Yes ☐ No
Cancer or other neoplastic syndrome	☐ Yes ☐ No
Ischemic Heart Disease	☐ Yes ☐ No
Cardiac or vascular surgery-	☐ Yes ☐ No

Diabetes and Foot ulceration Study		
Unique Identification Number:	UR:	Name:
DOB:	Initials:	

Congestive Heart Failure	☐ Yes ☐ No
Chronic Pulmonary Disease	☐ Yes ☐ No
Chronic Liver Disease	☐ Yes ☐ No
Chronic Renal Impairment	☐ Yes ☐ No
DVT- Deep vein thrombosis	Yes ☐ No
Charcot's neuroarthropathy/ Charcot foot	Yes ☐ No
Visual impairment from Macular degeneration or Glaucoma/ other	Yes ☐ No

Diabetes and ulcer history

Year and month Diagnosed-<br><br>Duration_____________years and _____________ months.	Uses insulin as a part of diabetes management?<br>Yes No
Positive Family History Yes No	HAB1C-
Have you had a foot ulcer in the past?<br>Yes No	Has this ulcer been plantar in location?<br>Yes No
Is the present ulcer in the same location where you had the last ulcer?<br>Yes No	Have you had more than one previous ulcer in the past?<br>Yes No

Physical Activity History

1. How many hours a week would you be standing? (work, home, leisure)<br><br>_________________________hrs	2. How many hours a week are you on your feet and walking?<br><br>___________________________hrs
3. Do you do regular exercise that includes walking?<br><br>Yes No	4. Do you get pain in your feet, legs or buttock areas that keep you from walking regularly?<br>Yes No
3A. If yes how many hours a week walking is this?<br>______________________ hrs.	5. How many hours a week are you off your feet (resting, sleeping, sitting<br><br>___________________hrs.

Total hours in three months standing (weight bearing)
_________________ hrs

Total hours in three months walking (weight bearing)
____________________________hrs

**Medication**	**Use**	**Daily Dose**

Footwear Assessment

What type of shoe do you currently use most of the time?

1. Sports   2. Dress   3.Diabetic special shoe   4.Custom made shoe   5.Thongs 6.None

On average per week how many days do you use this shoe?

Days out of seven? **/7**

Do you wear orthoses? **Yes   No   Custom   Off-the shelf**

Diabetes and Foot ulceration Study		
Unique Identification Number:	UR:	Name:
DOB:	Initials:	

Physical Measurements

Height: □□□cm	Weight: □□□□kg
BMI: ____________________________	
Waist circumference _________________cm	
Hip circumference ________________cm	

Visual test (Diabetic retinopathy)

Has the patient seen an optometrist in the last 12 months? Yes No

Optometrist Details-

Does the patient need optometrist referral?

Visual acuity score-

Podiatric Examination- To be conducted using a goniometer for values for ROM

**Examination**	**Right**	**Left**
Ankle Joint ROM (deg)		
STJ ROM (deg)		
1st MTPJ ROM (deg)		
HAV- Hallux Abducto Valgus Deformity Stage Manchester Scale		
Foot type	Pes Planus<br>Pes Cavus<br>Regular Arch Contour	Pes Planus<br>Pes Cavus<br>Regular Arch Contour
Muscle strength DF/PF	1 2 3<br>4 5	1 2 3<br>4 5
Muscle Strength Inv/Evr	1 2 3<br>4 5	1 2 3<br>4 5
Muscle Strength Abduction/ Adduction	1 2 3<br>4 5	1 2 3<br>4 5

Lesser toe deformities (Claw toe, Hammer Toe, Mallet toe)	Claw Toe<br>Hammer Toe<br>Mallet Toe	Claw Toe<br>Hammer Toe<br>Mallet Toe
Plantar hyperkeratosis locations	Plantar first metatarsal head<br>Plantar PMA 1-3<br>Plantar Apex of toes<br>Plantar Calcaneal area<br>Medial Hallux<br>Plantar 5th metatarsal head<br>Plantar PMA 4-5<br>Plantar Cuboid	Plantar first metatarsal head<br>Plantar PMA 1-3<br>Plantar Apex of toes<br>Plantar Calcaneal area<br>Medial Hallux<br>Plantar 5th metatarsal head<br>Plantar PMA 4-5<br>Plantar Cuboid
Lunge test	<35 Degrees<br>35 Degrees<br>>35 Degrees	<35 Degrees<br>35 Degrees<br>>35 Degrees
MSRT	1 2 3<br>4 5	1 2 3<br>4 5
Jack's test	Positive<br>Negative	Positive<br>Negative

Vascular and Neurological examination

Investigation	Right	Left
**Foot Pulses**<br>Posterior Tibialis<br>Dorsalis Pedis<br>Anterior tibialis	 Present/Normal Reduced Absent<br>Present/Normal Reduced Absent<br>Present/Normal Reduced Absent	 Present/Normal Reduced Absent<br>Present/Normal Reduced Absent<br>Present/Normal Reduced Absent
**ABI**<br>Brachial<br>Posterior Tibialis<br>Dorsalis Pedis<br>Anterior Tibial	 1<br>2<br>3<br>4<br>**ABI =**	 1<br>2<br>3<br>4<br>**ABI =**
Toe perfusion pressure (Hallux Pressure)	1<br>2<br>3<br>**Mean=**	1<br>2<br>3<br>**Mean=**
Monofilament test<br>Site specification (/10)	1. Plantar Hallux<br>2. Plantar metatarsal 2<br>3. Plantar metatarsal 3<br>4. Plantar metatarsal 4<br>5. Plantar metatarsal 5<br>6. Plantar Arch- (Navicular)<br>7. Plantar 2nd toe apex	1. Plantar Hallux<br>2. Plantar metatarsal 2<br>3. Plantar metatarsal 3<br>4. Plantar metatarsal 4<br>5. Plantar metatarsal 5<br>6. Plantar Arch- (Navicular)<br>7. Plantar 2nd toe apex

	8. Plantar 5th toe apex<br>9. Dorsal Hallux<br>10. Plantar Medial Tubercle	8. Plantar 5th toe apex<br>9. Dorsal Hallux<br>10. Plantar Medial Tubercle
Neurothesiometer Reading at which vibration felt at tip of hallux.	>25 V<br>25 V<br>< 25 V	>25 V<br>25 V<br>< 25 V

Does the patient have a present plantar foot ulcer?

Yes No

(If yes, please fill out planimetry and ulcer assessment table below and also get consent for biopsy)

Planimetry and Ulcer assessment

**Investigation**	**Ulcer 1**	**Ulcer 2**	**Ulcer 3**
Ulcer Location			
Estimated duration of ulcer<br>In weeks			
Planimetric depth cm3			
Planimetry<br>Length cm<br>Width cm<br>Area cm	Length _______cm<br>Width_______ cm<br>Area_______cm	Length _______cm<br>Width_______ cm<br>Area_______cm	Length _______cm<br>Width_______ cm<br>Area_______cm
Wound exudate/ discharge present and type of exudate	Mild Serous<br>Moderate<br>Purulent<br>High<br>Haemoserous	Mild Serous<br>Moderate Purulent<br>High<br>Haemoserous	Mild<br>Serous<br>Moderate<br>Purulent<br>High<br>Haemoserous
UTWCS Grading			
Wound Bed	Necrotic<br>Granulating<br>Epithelializing<br>Sloughy<br>Pale	Necrotic<br>Granulating<br>Epithelializing<br>Sloughy<br>Pale	Necrotic<br>Granulating<br>Epithelializing<br>Sloughy<br>Pale

Diabetes and Foot ulceration Study		
Unique Identification Number:	UR:	Name:
DOB:	Initials:	

	Hypergranulating Bone	Hypergranulating Bone	Hypergranulating Bone
Wound Edge	Regular<br>Irregular<br>Undermined<br>Rolling	Regular<br>Irregular<br>Undermined<br>Rolling	Regular<br>Irregular<br>Undermined<br>Rolling
Sinus Formation<br>Yes No	Yes No<br>mm____________	Yes No<br>mm ________________	Yes No<br>mm<br>________________
Is the ulcer infected? -Conduct Wound Swab for presence of pathological microbes.	Yes<br><br>No	Yes<br><br>No	Yes<br><br>No
Type of organism causing infection (As per pathology test)			
Type of ulcer (Neuropathic, Neuroischemic)	Neuropathic<br><br>Neuroischemic	Neuropathic<br><br>Neuroischemic	Neuropathic<br><br>Neuroischemic
Surrounding Skin	Macerated<br>Hyperkeratotic<br>Indurated<br>Normal/healthy<br>Fragile<br>Erythematous<br>Oedematous<br>Anhidrotic	Macerated<br>Hyperkeratotic<br>Indurated<br>Normal/healthy<br>Fragile<br>Erythematous<br>Oedematous<br>Anhidrotic	Macerated<br>Hyperkeratotic<br>Indurated<br>Normal/healthy<br>Fragile<br>Erythematous<br>Oedematous<br>Anhidrotic
Orthoses Used?	Yes No	Yes No	Yes No
Offloading shoe or boot-walker used?	Yes No	Yes No	Yes No
Type of Off-loading Device	TCC<br>Foot Orthoses<br>AFO<br>Padding<br>No offloading	TCC<br>Foot Orthoses<br>AFO<br>Padding<br>No offloading	TCC<br>Foot Orthoses<br>AFO<br>Padding<br>No offloading
How often is the ulcer seen by a health professional in a month? (X 3)			

How often is the ulcer dressing changed per month? (x 3)			

Is a photograph of the ulcer taken? Yes No

Note

Quality of life questionnaire to be handed to the patient at this point to fill out then the details and dates for gait assessment visit and follow-up visit on a follow-up card should be given to the patient. Contact details are then recorded separately.

Pedometer Given to Patient? Yes No

Blood Collection – Pathology

Refer patient to SNP pathology for blood collection.

Blood collection form given to patient?	☐ Yes ☐ No
Date of Collection	☐☐/☐☐☐/☐☐☐☐ (dd/mm/yyyy)
Haemoglobin ☐☐☐g/L	LD ☐☐☐U/L
WCC ☐☐.☐10^9/L	Cholesterol ☐.☐mmol/L
Platelets ☐☐☐10^9/L	Triglyceride ☐.☐mmol/L
Fibrinogen ☐.☐☐g/L	HDL ☐.☐mmol/L
Sodium ☐☐☐ mmol/L	LDL ☐.☐mmol/L
Potassium ☐.☐ mmol/L	CRP ☐☐☐mg/L
Urea ☐☐☐ mmol/L	Chloride ☐☐☐mmol/L
Creatinine ☐☐☐ µmol/L	Bicarbonate ☐☐mmol/L

Diabetes and Foot ulceration Study		
Unique Identification Number:	UR:	Name:
DOB:	Initials:	

eGFR □□□mL/min/1.73m^2	Calcium □.□□mmol/L
Fasting Glucose □□.□µmol/L	Phosphorus □.□mmol/L
HbA1C _______________%	Total protein □□g/L
CRP __________mg/L	Hematocrit □.□□mg/dL
ESR ____________mm/hr	Neuts □.□10^9/L
Parathyroid Hormone _________pg/ml	Lymphs □.□10^9/L
**N-(carboxymethyl) lysine (CML)** _____________________	Monos □.□10^9/L
**Non-CML advanced glycation end products (AGEs)** ________________	Eos □.□10^9/L
**Pentosidine** ____________________________	Baso □.□□10^9/L
**Homocystine**	

Vascular Biology Unit Blood Sample Storage Form

Blood Collection –For VBU Biomarker Staff to complete

Has blood been collected for study analysis? (Check with SNP and Ratnesh)	☐ Yes ☐ No
Date of Collection	☐☐/☐☐☐/☐☐☐☐ (dd/mm/yyyy)
2 x SST	☐ Yes ☐ No
2 x EDTA	☐ Yes ☐ No
1 x Sodium Citrate	☐ Yes ☐ No

Sample Storage

Have study bloods been processed and stored according to the lab manual?	☐ Yes ☐ No* *If NO, record a Protocol Deviation
Date of Processing	☐☐/☐☐☐/☐☐☐☐ (dd/mm/yyyy)

	Number of Samples:	Have samples been stored at the Protocol defined temperature?	Location of Samples
Serum<br>(from SST tubes)	☐☐<br>(Store at -80°C)	☐ Yes ☐ No*<br>*If NO, record a Protocol Deviation	Box #<br>Position #
EDTA Plasma<br>(Purple top EDTA tubes)	☐☐<br>(Store at -80°C)	☐ Yes ☐ No*<br>*If NO, record a Protocol Deviation	Box #<br>Position #
EDTA RBC Pellet<br>(Remaining pellet from EDTA tubes with plasma removed)	☐☐<br>(Store at -80°C)	☐ Yes ☐ No*<br>*If NO, record a Protocol Deviation	Box #<br>Position #
Citrate Plasma<br>(Blue top Citrate tubes)	☐☐<br>(Store at -80°C)	☐ Yes ☐ No*<br>*If NO, record a Protocol Deviation	Box #<br>Position #

Diabetes and Foot ulceration Study		
Unique Identification Number:	UR:	Name:
DOB:	Initials:	

Patient has completed blood test	☐ Yes ☐ No
Patient fulfils the criteria for the study?	☐ Yes ☐ No
Patient is going to be placed in the group	Case<br><br>Control
Patient has filled out a quality of life questionnaire/s	☐ Yes ☐ No
Patient has been advised about the gait assessment date and time and location of the gait lab	☐ Yes ☐ No
Patient has been advised of the date of next visit and advised to come after fasting for this, as if having a blood test	☐ Yes ☐ No

Patient Contact information for follow-up visits

**Name**
**Unique Identification Number -**
**Telephone number**
**Address**
**Email**
**Gait Assessment date and time -**
**Next estimated visit date (3 months) -**

Prior to Commencing Gait assessment
Claudication Questionnaire Checked? Yes† No
ABI is within normal range for participation? Yes No
Need vascular review? Yes No
Needs ulcer dressing? Yes No

Notes:

Visit 1 – Part 2: Gait, Pressure and Biomechanical Assessment.

Site- James Cook University, Gait Laboratory.

Physical Measurements

ASIS Breadth cm	Mass: □□□□ kg
Left Leg length cm	Height cm
Left Knee diameter cm	
Left malleolus width cm	
Right Leg length cm	
Right Knee diameter cm	
Right malleolus width cm	

Testing protocol

Measure up: ❑

EMG prep: ❑

MVC: ❑

Reflective markers prep ❑

Static capture ❑

10 walking trails ❑

Treadmill walking 4 minutes ❑

(Capture only 2:00 onwards)s
Self-Selected Walking Speed ______________________km/hr.

Plantar pressure capture (3 walks) ❑

FINISHED

Data-Sets Post-Data extraction

**Primary Outcome measure**	**Measurement**	**Left**	**Right**
Peak plantar pressure (N/Cm2)	Medial Heel-<br>Lateral Heel<br>Medial Forefoot<br>Lateral Forefoot		
Pressure time integral (N/Cm2) X s	Medial Heel-<br>Lateral Heel<br>Medial Forefoot<br>Lateral Forefoot		
Time of peak occurrence EMG (%)	Vastus Lateralis<br>Lat Gastrocnemius<br>Tibialis Anterior		
GRF (N/Kg)^D	Vertical<br>Med/Lat<br>Ant/Post		
Oxford Foot Model (deg)	Hindfoot Sag<br>Hindfoot Fron<br>Hindfoot T.V<br>Forefoot Sag<br>Forefoot Fron<br>Forefoot T.V		
Lower Limb Kinematics	Stance Phase<br>Max hip Flex<br>Max hip Ext<br>Max Knee Flex<br>Max Knee Ext<br>Max Ankle DF<br>Max Ankle PF<br><br>Swing Phase<br>Max hip Flex<br>Max hip Ext<br>Max Knee Flex<br>Max Knee Ext<br>Max Ankle DF<br>Max Ankle PF		

EMG During Gait Assessment

Maximum Voluntary Contraction

Tibialis Anterior	Medial Gastroc	Lateral Gastroc	Peroneus Longus	Soleus	VMO	Semiten
Right						
Left						

(%)	Tibialis Anterior	Medial Gastroc	Lateral Gastroc	Peroneus Longus	Soleus	VMO	Semiten
Peak Activation<br>Left<br>Right							
Contact<br>Left<br>Right							
Stance<br>Left<br>Right							
Toe-off<br>Left<br>Right							
Swing<br>Left<br>Right							

Treadmill gait assessment

Assessment	Parameter	Left	Right
Oxford Foot Model (deg)	Hindfoot Sag<br>Hindfoot Fron<br>Hindfoot T.V<br>Forefoot Sag<br>Forefoot Fron<br>Forefoot T.V		
Lower Limb Kinematics	**Stance Phase**<br>Max hip Flex<br>Max hip Ext<br>Max Knee Flex<br>Max Knee Ext<br>Max Ankle DF<br>Max Ankle PF<br><br>**Swing Phase**<br>Max hip Flex<br>Max hip Ext<br>Max Knee Flex<br>Max Knee Ext<br>Max Ankle DF<br>Max Ankle PF		

EMG During Treadmill Gait

Muscle	Tibialis Anterior	Medial Gastroc	Lateral Gastroc	Peroneus Longus	Soleus	VMO	Semitendinosus
Peak Activation<br>Left<br>Right							
MVC<br>Left<br>Right							
Contact<br>Left<br>Right							
Stance<br>Left							

Right							
<u>Toe-off</u><br>Left<br>Right							
<u>Swing</u><br>Left<br>Right							

1. Blood Collection Form Given

2. Questionnaires Collected (4)

3. Review-date organised

4. Pedometer calibrated and given?

5. Instructions given on Pedometer return?

<u>Notes</u>

Visit 2
At 3 Months –Townsville hospital Diabetes clinic

Initial assessment

1	Is the participant in the case or control group?	Case<br><br>Control
2	Does the participant have a present plantar foot ulcer?	Yes ☐ No
3	Does the participant have a newly formed plantar ulcer?	Yes ☐ No
3A	Has a plantar ulcer healed in this duration?	**Yes No**
4	Does the participant give consent for biopsy of the ulcer/ulcers?	☐ Yes ☐ No
5	Has the participant's diabetes medication or dose changed since last visit?<br>Please note changes:<br>1.<br>2.<br>3.<br>4.<br>5.	☐ Yes ☐ No
6	Has the participant increased/decreased treatment frequency for the ulcer?	☐ Yes ☐ No
	If yes, how so?	Increased<br><br>Decreased
7	Has the patient commenced any new treatment for a disease process or as a part of a health measure?<br>I.e.- Dialysis, HRT, Warfarin treatment, Radiation therapy	☐ Yes ☐ No
	Please state ____	
8	Has the patient commenced or is awaiting any surgical procedure?	☐Yes ☐ No
	Please state ____	
9	Has the patient increased or decreased weight-bearing activity levels in the last 3 months?	☐Yes ☐ No
	Please state new number of hours per week, on weight-bearing activities?<br>__________________hrs	

Diabetes and Foot ulceration Study		
Unique Identification Number:	UR:	Name:
DOB:	Initials:	

	Please state the new number of hours per week weight-bearing but not doing any activity (i.e. standing) _____________________hrs<br><br>Please state the number of hours completely not weight bearing (Sitting) ______________________________hrs
**Total**	Total hours in three months standing (weight bearing) __________________ hrs<br><br>Total hours in three months walking (weight bearing) _____________________________hrs<br><br>Total hours in three months non-weight bearing _______________________________hrs

Physical Measurements

Weight: □ □ □ □ kg	Waist circumference _________________cm
BMI: ____________________________	Hip circumference ________________cm

Podiatric Examination- To be conducted using a goniometer for values for ROM

**Examination**	**Right**	**Left**
Ankle Joint ROM (deg)		
STJ ROM (deg)		
1st MTPJ ROM (deg)		
HAV- Hallux Abducto Valgus Deformity Stage Manchester Scale		
Foot type	Pes Planus<br>Pes Cavus<br>Regular Arch Contour	Pes Planus<br>Pes Cavus<br>Regular Arch Contour
Muscle strength DF/PF	1 2 3<br>4 5	1 2 3<br>4 5
Muscle Strength Inv/Evr	1 2 3<br>4 5	1 2 3<br>4 5
Muscle Strength Abduction/ Adduction	1 2 3<br>4 5	1 2 3<br>4 5

Lesser toe deformities (Claw toe, Hammer Toe, Mallet toe)	Claw Toe<br>Hammer Toe<br>Mallet Toe	Claw Toe<br>Hammer Toe<br>Mallet Toe
Plantar hyperkeratosis locations	Plantar first metatarsal head<br>Plantar PMA 1-3<br>Plantar Apex of toes<br>Plantar Calcaneal area<br>Medial Hallux<br>Plantar 5th metatarsal head<br>Plantar PMA 4-5<br>Plantar Cuboid	Plantar first metatarsal head<br>Plantar PMA 1-3<br>Plantar Apex of toes<br>Plantar Calcaneal area<br>Medial Hallux<br>Plantar 5th metatarsal head<br>Plantar PMA 4-5<br>Plantar Cuboid
Lunge test	<35 Degrees<br>35 Degrees<br>>35 Degrees	<35 Degrees<br>35 Degrees<br>>35 Degrees
MSRT	1 2 3<br>4 5	1 2 3<br>4 5
Jack's test	Positive<br>Negative	Positive<br>Negative

Does the patient have a present plantar foot ulcer?

Yes No

If yes, please fill out planimetry and ulcer assessment table below.

Planimetry and Ulcer assessment

**Investigation**	**Ulcer 1**	**Ulcer 2**	**Ulcer 3**
Ulcer Location			
Estimated duration of ulcer<br>In weeks			
Planimetric depth cm3			
Planimetry<br>Length cm<br>Width cm<br>Area cm	Length _______cm<br>Width_______ cm<br>Area_______cm	Length _______cm<br>Width_______ cm<br>Area_______cm	Length _______cm<br>Width_______ cm<br>Area_______cm
Wound exudate/ discharge present and type of exudate	Mild Serous<br>Moderate<br>Purulent	Mild Serous<br>Moderate Purulent<br>High	Mild<br>Serous<br>Moderate

	High<br>Haemoserous	Haemoserous	Purulent<br>High<br>Haemoserous
UTWCS Grading			
Wound Bed	Necrotic<br>Granulating<br>Epithelializing<br>Sloughy<br>Pale<br>Hypergranulating<br>Bone	Necrotic<br>Granulating<br>Epithelializing<br>Sloughy<br>Pale<br>Hypergranulating<br>Bone	Necrotic<br>Granulating<br>Epithelializing<br>Sloughy<br>Pale<br>Hypergranulating<br>Bone
Wound Edge	Regular<br>Irregular<br>Undermined<br>Rolling	Regular<br>Irregular<br>Undermined<br>Rolling	Regular<br>Irregular<br>Undermined<br>Rolling
Sinus Formation<br>Yes No	Yes No<br>mm___________	Yes No<br>mm _______________	Yes No<br>mm<br>_______________
Is the ulcer infected? -Conduct Wound Swab for presence of pathological microbes.	Yes<br><br>No	Yes<br><br>No	Yes<br><br>No
Type of organism causing infection (As per pathology test)			
Type of ulcer (Neuropathic, Neuroischemic)	Neuropathic<br><br>Neuroischemic	Neuropathic<br><br>Neuroischemic	Neuropathic<br><br>Neuroischemic
Surrounding Skin	Macerated<br>Hyperkeratotic<br>Indurated<br>Normal/healthy<br>Fragile<br>Erythematous<br>Oedematous<br>Anhidrotic	Macerated<br>Hyperkeratotic<br>Indurated<br>Normal/healthy<br>Fragile<br>Erythematous<br>Oedematous<br>Anhidrotic	Macerated<br>Hyperkeratotic<br>Indurated<br>Normal/healthy<br>Fragile<br>Erythematous<br>Oedematous<br>Anhidrotic
Orthoses Used?	Yes No	Yes No	Yes No
Offloading shoe or boot-walker used?	Yes No	Yes No	Yes No

Diabetes and Foot ulceration Study		
Unique Identification Number:	UR:	Name:
DOB:	Initials:	

Type of Off-loading Device	TCC<br>Foot Orthoses<br>AFO<br>Padding<br>No offloading	TCC<br>Foot Orthoses<br>AFO<br>Padding<br>No offloading	TCC<br>Foot Orthoses<br>AFO<br>Padding<br>No offloading
How often is the ulcer seen by a health professional in a month? (X 3)			
How often is the ulcer dressing changed per month? (x 3)			

<u>Is a photograph of the ulcer taken?</u> Yes No

<u>Footwear Assessment</u>

What type of shoe do you currently use most of the time?

1. Sports 2. Dress 3.Diabetic special shoe 4.Custom made shoe 5.Thongs 6.None

On average per week how many days do you use this shoe?

Days out of seven? **/7**

Do you wear orthoses? **Yes No Custom Off-the shelf**

<u>Blood Collection – Pathology</u>

Refer patient to SNP pathology for blood collection.

Blood collection form given to patient?	□ Yes □ No
Date of Collection	□□/□□□/□□□□ (dd/mm/yyyy)
Haemoglobin □□□g/L	LD □□□U/L
WCC □□.□10^9/L	Cholesterol □.□mmol/L
Platelets □□□10^9/L	Triglyceride □.□mmol/L
Fibrinogen □.□□g/L	HDL □.□mmol/L
Sodium □□□ mmol/L	LDL □.□mmol/L

Potassium □.□ mmol/L	CRP □□□mg/L
Urea □□□ mmol/L	Chloride □□□mmol/L
Creatinine □□□ µmol/L	Bicarbonate □□mmol/L
eGFR □□□mL/min/1.73m^2	Calcium □.□□mmol/L
Fasting Glucose □□.□µmol/L	Phosphorus □.□mmol/L
HAB1C ________________%	Total protein □□g/L
CRP __________mg/L	Hematocrit □.□□mg/dL
ESR ____________mm/hr	Neuts □.□10^9/L
Parathyroid Hormone _________pg/ml	Lymphs □.□10^9/L
**N-(carboxymethyl) lysine (CML)** ______________________	Monos □.□10^9/L
**Non-CML advanced glycation end products (AGEs)** _________________	Eos □.□10^9/L
**Pentosidine** ______________________________	Baso □.□□10^9/L
**Homocystine**	

Note

Quality of life questionnaire to be handed to the patient at this point to fill out then the details and dates for gait assessment visit and follow-up visit on a follow-up card should be given to the patient. Contact details are then recorded separately.

Pedometer Given to Patient? Yes No

Diabetes and Foot ulceration Study		
Unique Identification Number:	UR:	Name:
DOB:	Initials:	

Vascular Biology Unit Blood Sample Storage Form

Blood Collection –For VBU Biomarker Staff to complete

Has blood been collected for study analysis? (Check with SNP and Ratnesh)	☐ Yes ☐ No
Date of Collection	☐☐/☐☐☐/☐☐☐☐ (dd/mm/yyyy)
2 x SST	☐ Yes ☐ No
2 x EDTA	☐ Yes ☐ No
1 x Sodium Citrate	☐ Yes ☐ No

Sample Storage

Have study bloods been processed and stored according to the lab manual?		☐ Yes ☐ No* *If NO, record a Protocol Deviation	
Date of Processing		☐☐/☐☐☐/☐☐☐☐ (dd/mm/yyyy)	
	Number of Samples:	Have samples been stored at the Protocol defined temperature?	Location of Samples
Serum (from SST tubes)	☐☐ (Store at -80°C)	☐ Yes ☐ No* *If NO, record a Protocol Deviation	Box # Position #
EDTA Plasma (Purple top EDTA tubes)	☐☐ (Store at -80°C)	☐ Yes ☐ No* *If NO, record a Protocol Deviation	Box # Position #
EDTA RBC Pellet (Remaining pellet from EDTA tubes with plasma removed)	☐☐ (Store at -80°C)	☐ Yes ☐ No* *If NO, record a Protocol Deviation	Box # Position #
Citrate Plasma (Blue top Citrate tubes)	☐☐ (Store at -80°C)	☐ Yes ☐ No* *If NO, record a Protocol Deviation	Box # Position #

Notes:

Biopsy Procedure Documentation Sheet- Foot ulcer patients Only!

Has informed consent been obtained for biopsy? Yes No

Have VBU staff been advised about time of biopsy collection? Yes No

Appropriate transport medium organised? Yes No

Doctors Statement

I have explained

- The patient's condition
- Need for the biopsy
- The procedure and the risk
- Relevant treatment options and their risks
- Likely consequences if those risks occur
- The significant risks and problems specific to this patient.

I have given the patient/substitute decision maker an opportunity to

- Ask questions about any of the above matters
- Raise any other concerns

Which I have answered as fully as possible. I am of the opinion that the patient/ substitute decision maker understands the above information.

Name of Doctor

____

Designation

____

Date

____

Signature

____

Biopsy Sample appropriately stored at the VBU? Yes No

Date of storage of Sample-

Part 2: Gait, Pressure and Biomechanical Assessment.

Site- James Cook University, Gait Laboratory.

Physical Measurements

ASIS Breadth cm	Mass: ☐ ☐ ☐ ☐ kg
Left Leg length cm	Height cm
Left Knee diameter cm	
Left malleolus width cm	
Right Leg length cm	
Right Knee diameter cm	
Right malleolus width cm	

Testing protocol

Measure up: ❑

EMG prep: ❑

MVC: ❑

Reflective markers prep ❑

Static capture ❑

10 walking trails ❑

Treadmill walking 4 minutes ❑

(Capture only 2:00 onwards)s
Self-Selected Walking Speed ______________________km/hr.

Plantar pressure capture (3 walks) ❑

FINISHED

**Primary Outcome measure**	**Measurement**	**Left**	**Right**
Peak plantar pressure (N/Cm2)	Medial Heel-<br>Lateral Heel<br>Medial Forefoot<br>Lateral Forefoot		
Pressure time integral (N/Cm2) X s	Medial Heel-<br>Lateral Heel<br>Medial Forefoot<br>Lateral Forefoot		
Time of peak occurrence EMG (%)	Vastus Lateralis<br>Lat Gastrocnemius<br>Tibialis Anterior		
GRF (N/Kg)^D	Vertical<br>Med/Lat<br>Ant/Post		
Oxford Foot Model (deg)	Hindfoot Sag<br>Hindfoot Fron<br>Hindfoot T.V<br>Forefoot Sag<br>Forefoot Fron<br>Forefoot T.V		
Lower Limb Kinematics	Stance Phase<br>Max hip Flex<br>Max hip Ext<br>Max Knee Flex<br>Max Knee Ext<br>Max Ankle DF<br>Max Ankle PF<br><br>Swing Phase<br>Max hip Flex<br>Max hip Ext<br>Max Knee Flex<br>Max Knee Ext<br>Max Ankle DF<br>Max Ankle PF		

Data-Sets Post-Data extraction

EMG During Gait Assessment

Maximum Voluntary Contraction

Tibialis Anterior	Medial Gastroc	Lateral Gastroc	Peroneus Longus	Soleus	VMO	Semiten
Right						
Left						

(%)	Tibialis Anterior	Medial Gastroc	Lateral Gastroc	Peroneus Longus	Soleus	VMO	Semiten
Peak Activation Left Right							
Contact Left Right							
Stance Left Right							
Toe-off Left Right							
Swing Left Right							

Treadmill gait assessment

Assessment	Parameter	Left	Right

Oxford Foot Model (deg)	Hindfoot Sag<br>Hindfoot Fron<br>Hindfoot T.V<br>Forefoot Sag<br>Forefoot Fron<br>Forefoot T.V		
Lower Limb Kinematics	**Stance Phase**<br>Max hip Flex<br>Max hip Ext<br>Max Knee Flex<br>Max Knee Ext<br>Max Ankle DF<br>Max Ankle PF<br><br>**Swing Phase**<br>Max hip Flex<br>Max hip Ext<br>Max Knee Flex<br>Max Knee Ext<br>Max Ankle DF<br>Max Ankle PF		

EMG During Treadmill Gait

Muscle	Tibialis Anterior	Medial Gastroc	Lateral Gastroc	Peroneus Longus	Soleus	VMO	Semitendinosus
Peak Activation<br>Left<br>Right							
MVC<br>Left<br>Right							
Contact<br>Left<br>Right							
Stance<br>Left<br>Right							

Toe-off<br>Left<br>Right							
Swing<br>Left<br>Right							

Summary of Findings

Is the initial ulcer still present?	**YES**	**NO**
Has the ulcer size increased?	**YES**	**NO**
Has the ulcer size decreased?	**YES**	**NO**
Is there formation of a new ulcer?	**YES**	**NO**
Has the ulcer completely healed?	**YES**	**NO**
Is there a decrease in glycaemic control?	**YES**	**NO**
Is there a decrease in renal function?	**YES**	**NO**
Has the patient commenced dialysis?	**YES**	**NO**
Is there increased AGE formation present?	**YES**	**NO**
Are there differences in the ROM of joints?	**YES**	**NO**
Is there deterioration of vascular function?	**YES**	**NO**
Is there a deterioration of neurological function?	**YES**	**NO**
Has there been a reduction in treatment frequency?	**YES**	**NO**
Has there been an increase in treatment frequency?	**YES**	**NO**
Was a biopsy conducted?	**YES**	**NO**
Is there infection present?	**YES**	**NO**
Has haematological status deteriorated critically? I.e.- Lipid profile, WCC, CRP, ESR	**YES**	**NO**
Is the participant still suitable for the study?	**YES**	**NO**
Next visit date provided?	**YES**	**NO**

If the participant is no longer suitable for the study, please send a letter of thanks for being involved in the study, otherwise provide date and time of next consultation.

Date and time of next consultation ___________________________

1. Blood Collection Form Given

2. Questionnaires Collected (4)

3. Review-date organised

4. Pedometer calibrated and given?

5. Instructions given on Pedometer return?

Notes

Visit 3
At 6 Months

Initial assessment

Diabetes and Foot ulceration Study		
Unique Identification Number:	UR:	Name:
DOB:	Initials:	

1	Is the participant in the case or control group?	Case<br>Control
2	Does the participant have a present plantar foot ulcer?	Yes ☐ No
3	Does the participant have a newly formed plantar ulcer?	Yes ☐ No
3A	Has a plantar ulcer healed in this duration?	**Yes No**
4	Does the participant give consent for biopsy of the ulcer/ulcers?	☐ Yes ☐ No
5	Has the participant's diabetes medication or dose changed since last visit?<br>Please note changes:<br>1.<br>2.<br>3.<br>4.<br>5.	☐ Yes ☐ No
6	Has the participant increased/decreased treatment frequency for the ulcer?	☐ Yes ☐ No
	If yes, how so?	Increased<br>Decreased
7	Has the patient commenced any new treatment for a disease process or as a part of a health measure?<br>I.e.- Dialysis, HRT, Warfarin treatment, Radiation therapy	☐ Yes ☐ No
	Please state ______________________________________	
8	Has the patient commenced or is awaiting any surgical procedure?	Yes ☐ No
	Please state ______________________________________	
9	Has the patient increased or decreased weight-bearing activity levels in the last 3 months?	Yes ☐ No
	Please state new number of hours per week, on weight-bearing activities?<br>_________________hrs<br><br>Please state the new number of hours per week weight-bearing but not doing any activity (ie standing)<br>______________________hrs	

	Please state the number of hours completely not weight bearing (Sitting)<br>______________________________hrs
**Total**	Total hours in three months standing (weight bearing)<br>__________________ hrs<br><br>Total hours in three months walking (weight bearing)<br>_____________________________hrs<br><br>Total hours in three months non-weight bearing<br>_______________________________hrs

Physical Measurements

Weight: ☐☐☐☐kg	Waist circumference<br>_________________cm
BMI: ____________________________	Hip circumference ________________cm

Podiatric Examination- To be conducted using a goniometer for values for ROM

**Examination**	**Right**	**Left**
Ankle Joint ROM (deg)		
STJ ROM (deg)		
1st MTPJ ROM (deg)		
HAV- Hallux Abducto Valgus Deformity Stage Manchester Scale		
Foot type	Pes Planus<br>Pes Cavus<br>Regular Arch Contour	Pes Planus<br>Pes Cavus<br>Regular Arch Contour
Muscle strength DF/PF	1 2 3<br>4 5	1 2 3<br>4 5
Muscle Strength Inv/Evr	1 2 3<br>4 5	1 2 3<br>4 5
Muscle Strength Abduction/ Adduction	1 2 3<br>4 5	1 2 3<br>4 5
Lesser toe deformities (Claw toe, Hammer Toe, Mallet toe)	Claw Toe<br>Hammer Toe<br>Mallet Toe	Claw Toe<br>Hammer Toe<br>Mallet Toe
Plantar hyperkeratosis locations	Plantar first metatarsal head<br>Plantar PMA 1-3	Plantar first metatarsal head<br>Plantar PMA 1-3

Diabetes and Foot ulceration Study		
Unique Identification Number:	UR:	Name:
DOB:	Initials:	

	Plantar Apex of toes<br>Plantar Calcaneal area<br>Medial Hallux<br>Plantar 5th metatarsal head<br>Plantar PMA 4-5<br>Plantar Cuboid	Plantar Apex of toes<br>Plantar Calcaneal area<br>Medial Hallux<br>Plantar 5th metatarsal head<br>Plantar PMA 4-5<br>Plantar Cuboid
Lunge test	<35 Degrees<br>35 Degrees<br>>35 Degrees	<35 Degrees<br>35 Degrees<br>>35 Degrees
MSRT	1 2 3<br>4 5	1 2 3<br>4 5
Jack's test	Positive<br>Negative	Positive<br>Negative

Does the patient have a present plantar foot ulcer?

Yes No

If yes, please fill out planimetry and ulcer assessment table below.

Planimetry and Ulcer assessment

**Investigation**	**Ulcer 1**	**Ulcer 2**	**Ulcer 3**
Ulcer Location			
Estimated duration of ulcer<br>In weeks			
Planimetric depth cm3			
Planimetry<br>Length cm<br>Width cm<br>Area cm	Length _______cm<br>Width_______ cm<br>Area_______cm	Length _______cm<br>Width_______ cm<br>Area_______cm	Length _______cm<br>Width_______ cm<br>Area_______cm
Wound exudate/ discharge present and type of exudate	Mild Serous<br>Moderate<br>Purulent<br>High<br>Haemoserous	Mild Serous<br>Moderate Purulent<br>High<br>Haemoserous	Mild<br>Serous<br>Moderate<br>Purulent<br>High<br>Haemoserous
UTWCS Grading			

Wound Bed	Necrotic<br>Granulating<br>Epithelializing<br>Sloughy<br>Pale<br>Hypergranulating<br>Bone	Necrotic<br>Granulating<br>Epithelializing<br>Sloughy<br>Pale<br>Hypergranulating<br>Bone	Necrotic<br>Granulating<br>Epithelializing<br>Sloughy<br>Pale<br>Hypergranulating<br>Bone
Wound Edge	Regular<br>Irregular<br>Undermined<br>Rolling	Regular<br>Irregular<br>Undermined<br>Rolling	Regular<br>Irregular<br>Undermined<br>Rolling
Sinus Formation<br>Yes No	Yes No<br>mm____________	Yes No<br>mm ________________	Yes No<br>mm<br>________________
Is the ulcer infected?<br>-Conduct Wound Swab for presence of pathological microbes.	Yes<br><br>No	Yes<br><br>No	Yes<br><br>No
Type of organism causing infection (As per pathology test)			
Type of ulcer (Neuropathic, Neuroischemic)	Neuropathic<br><br>Neuroischemic	Neuropathic<br><br>Neuroischemic	Neuropathic<br><br>Neuroischemic
Surrounding Skin	Macerated<br>Hyperkeratotic<br>Indurated<br>Normal/healthy<br>Fragile<br>Erythematous<br>Oedematous<br>Anhidrotic	Macerated<br>Hyperkeratotic<br>Indurated<br>Normal/healthy<br>Fragile<br>Erythematous<br>Oedematous<br>Anhidrotic	Macerated<br>Hyperkeratotic<br>Indurated<br>Normal/healthy<br>Fragile<br>Erythematous<br>Oedematous<br>Anhidrotic
Orthoses Used?	Yes No	Yes No	Yes No
Offloading shoe or boot-walker used?	Yes No	Yes No	Yes No
Type of Off-loading Device	TCC<br>Foot Orthoses<br>AFO<br>Padding<br>No offloading	TCC<br>Foot Orthoses<br>AFO<br>Padding<br>No offloading	TCC<br>Foot Orthoses<br>AFO<br>Padding<br>No offloading

How often is the ulcer seen by a health professional in a month? (X 3)			
How often is the ulcer dressing changed per month? (x 3)			

Footwear Assessment

What type of shoe do you currently use most of the time?

1. Sports 2. Dress 3.Diabetic special shoe 4.Custom made shoe 5.Thongs 6.None

On average per week how many days do you use this shoe?

Days out of seven? **/7**

Do you wear orthoses? **Yes No Custom Off-the shelf**

Note

Quality of life questionnaire to be handed to the patient at this point to fill out then the details and dates for gait assessment visit and follow-up visit on a follow-up card should be given to the patient. Contact details are then recorded separately.

Pedometer Given to Patient? Yes No

Blood Collection – Pathology

Refer patient to SNP pathology for blood collection.

Blood collection form given to patient?	☐ Yes ☐ No

Diabetes and Foot ulceration Study		
Unique Identification Number:	UR:	Name:
DOB:	Initials:	

Date of Collection	□□/□□□/□□□□ (dd/mm/yyyy)
Haemoglobin □□□g/L	LD □□□U/L
WCC □□.□10^9/L	Cholesterol □.□mmol/L
Platelets □□□10^9/L	Triglyceride □.□mmol/L
Fibrinogen □.□□g/L	HDL □.□mmol/L
Sodium □□□ mmol/L	LDL □.□mmol/L
Potassium □.□ mmol/L	CRP □□□mg/L
Urea □□□ mmol/L	Chloride □□□mmol/L
Creatinine □□□ µmol/L	Bicarbonate □□mmol/L
eGFR □□□mL/min/1.73m^2	Calcium □.□□mmol/L
Fasting Glucose □□.□µmol/L	Phosphorus □.□mmol/L
HAB1C ________________%	Total protein □□g/L
CRP __________mg/L	Hematocrit □.□□mg/dL
ESR ____________mm/hr	Neuts □.□10^9/L
Parathyroid Hormone _________pg/ml	Lymphs □.□10^9/L
**N-(carboxymethyl) lysine (CML)** ______________________	Monos □.□10^9/L
**Non-CML advanced glycation end products (AGEs)** _________________	Eos □.□10^9/L
**Pentosidine** ______________________________	Baso □.□□10^9/L
**Homocystine**	

Vascular Biology Unit Blood Sample Storage Form

Diabetes and Foot ulceration Study		
Unique Identification Number:	UR:	Name:
DOB:	Initials:	

Blood Collection –For VBU Biomarker Staff to complete

Has blood been collected for study analysis? (Check with SNP and Ratnesh)	☐ Yes ☐ No
Date of Collection	☐☐/☐☐☐/☐☐☐☐ (dd/mm/yyyy)
2 x SST	☐ Yes ☐ No
2 x EDTA	☐ Yes ☐ No
1 x Sodium Citrate	☐ Yes ☐ No

Sample Storage

Have study bloods been processed and stored according to the lab manual?		☐ Yes ☐ No* *If NO, record a Protocol Deviation	
Date of Processing		☐☐/☐☐☐/☐☐☐☐ (dd/mm/yyyy)	
	Number of Samples:	Have samples been stored at the Protocol defined temperature?	Location of Samples
Serum (from SST tubes)	☐☐ (Store at -80°C)	☐ Yes ☐ No* *If NO, record a Protocol Deviation	Box # Position #
EDTA Plasma (Purple top EDTA tubes)	☐☐ (Store at -80°C)	☐ Yes ☐ No* *If NO, record a Protocol Deviation	Box # Position #
EDTA RBC Pellet (Remaining pellet from EDTA tubes with plasma removed)	☐☐ (Store at -80°C)	☐ Yes ☐ No* *If NO, record a Protocol Deviation	Box # Position #
Citrate Plasma (Blue top Citrate tubes)	☐☐ (Store at -80°C)	☐ Yes ☐ No* *If NO, record a Protocol Deviation	Box # Position #

Diabetes and Foot ulceration Study		
Unique Identification Number:	UR:	Name:
DOB:	Initials:	

Additional Biopsy Procedure Documentation Sheet-

Foot ulcer patients only as clinically required.

Has informed consent been obtained for biopsy? Yes No

Has VBU staff been advised about time of biopsy collection? Yes No

Appropriate transport medium organised? Yes No

Doctors Statement

I have explained

- The patient's condition
- Need for the biopsy
- The procedure and the risk
- Relevant treatment options and their risks
- Likely consequences if those risks occur
- The significant risks and problems specific to this patient.

I have given the patient/substitute decision maker an opportunity to

- Ask questions about any of the above matters
- Raise any other concerns

Which I have answered as fully as possible. I am of the opinion that the patient/ substitute decision maker understands the above information.

Name of Doctor

____

Designation

____

Date

____

Signature

____

Biopsy Sample appropriately stored at the VBU? Yes No

Date of storage of Sample-

Part 2: Gait, Pressure and Biomechanical Assessment.

Diabetes and Foot ulceration Study		
Unique Identification Number:	UR:	Name:
DOB:	Initials:	

Site- James Cook University, Gait Laboratory.

Physical Measurements

ASIS Breadth cm	Mass: □ □ □ □ kg
Left Leg length cm	Height cm
Left Knee diameter cm	
Left malleolus width cm	
Right Leg length cm	
Right Knee diameter cm	
Right malleolus width cm	

Testing protocol

Measure up: ❑

EMG prep: ❑

MVC: ❑

Reflective markers prep ❑

Static capture ❑

10 walking trails ❑

Treadmill walking 4 minutes ❑

(Capture only 2:00 onwards)s
Self-Selected Walking Speed ______________________km/hr.

Plantar pressure capture (3 walks) ❑

FINISHED

**Primary Outcome measure**	**Measurement**	**Left**	**Right**
Peak plantar pressure (N/Cm2)	Medial Heel-<br>Lateral Heel<br>Medial Forefoot<br>Lateral Forefoot		
Pressure time integral (N/Cm2) X s	Medial Heel-<br>Lateral Heel<br>Medial Forefoot<br>Lateral Forefoot		
Time of peak occurrence EMG (%)	Vastus Lateralis<br>Lat Gastrocnemius<br>Tibialis Anterior		
GRF (N/Kg)^D	Vertical<br>Med/Lat<br>Ant/Post		
Oxford Foot Model (deg)	Hindfoot Sag<br>Hindfoot Fron<br>Hindfoot T.V<br>Forefoot Sag<br>Forefoot Fron<br>Forefoot T.V		
Lower Limb Kinematics	Stance Phase<br>Max hip Flex<br>Max hip Ext<br>Max Knee Flex<br>Max Knee Ext<br>Max Ankle DF<br>Max Ankle PF<br><br>Swing Phase<br>Max hip Flex<br>Max hip Ext<br>Max Knee Flex<br>Max Knee Ext<br>Max Ankle DF<br>Max Ankle PF		

Data-Sets Post-Data extraction

EMG During Gait Assessment

Maximum Voluntary Contraction

Tibialis Anterior	Medial Gastroc	Lateral Gastroc	Peroneus Longus	Soleus	VMO	Semiten
Right						
Left						

(%)	Tibialis Anterior	Medial Gastroc	Lateral Gastroc	Peroneus Longus	Soleus	VMO	Semiten
Peak Activation<br>Left<br>Right							
Contact<br>Left<br>Right							
Stance<br>Left<br>Right							
Toe-off<br>Left<br>Right							
Swing<br>Left<br>Right							

Treadmill gait assessment

Assessment	Parameter	Left	Right

Oxford Foot Model (deg)	Hindfoot Sag<br>Hindfoot Fron<br>Hindfoot T.V<br>Forefoot Sag<br>Forefoot Fron<br>Forefoot T.V		
Lower Limb Kinematics	**Stance Phase**<br>Max hip Flex<br>Max hip Ext<br>Max Knee Flex<br>Max Knee Ext<br>Max Ankle DF<br>Max Ankle PF<br><br>**Swing Phase**<br>Max hip Flex<br>Max hip Ext<br>Max Knee Flex<br>Max Knee Ext<br>Max Ankle DF<br>Max Ankle PF		

EMG During Treadmill Gait

Muscle	Tibialis Anterior	Medial Gastroc	Lateral Gastroc	Peroneus Longus	Soleus	VMO	Semitendinosus
Peak Activation<br>Left<br>Right							
MVC<br>Left<br>Right							
Contact<br>Left<br>Right							
Stance<br>Left<br>Right							

Toe-off<br>Left<br>Right							
Swing<br>Left<br>Right							

Summary of Findings

Is the initial ulcer still present?	**YES**	**NO**
Has the ulcer size increased?	**YES**	**NO**
Has the ulcer size decreased?	**YES**	**NO**
Is there formation of a new ulcer?	**YES**	**NO**
Has the ulcer completely healed?	**YES**	**NO**
Is there a decrease in glycaemic control?	**YES**	**NO**
Is there a decrease in renal function?	**YES**	**NO**
Has the patient commenced dialysis?	**YES**	**NO**
Is there increased AGE formation present?	**YES**	**NO**
Are there differences in the ROM of joints?	**YES**	**NO**
Is there deterioration of vascular function?	**YES**	**NO**
Is there a deterioration of neurological function?	**YES**	**NO**
Has there been a reduction in treatment frequency?	**YES**	**NO**
Has there been an increase in treatment frequency?	**YES**	**NO**
Was a biopsy conducted?	**YES**	**NO**
Is there infection present?	**YES**	**NO**
Has haematological status deteriorated critically? I.e.- Lipid profile, WCC, CRP, ESR	**YES**	**NO**
Is the participant still suitable for the study?	**YES**	**NO**
Next visit date provided?	**YES**	**NO**

If the participant is no longer suitable for the study, please send a letter of thanks for being involved in the study, otherwise provide date and time of next consultation.

Date and time of next consultation ___________________________

1. Blood Collection Form Given

2. Questionnaires Collected (4)

3. Review-date organised

4. Pedometer calibrated and given?

5. Instructions given on Pedometer return?

Notes

Summary of Findings

Diabetes and Foot ulceration Study		
Unique Identification Number:	UR:	Name:
DOB:	Initials:	

Is the same ulcer still present?	**YES**	**NO**
Has the ulcer size increased?	**YES**	**NO**
Has the ulcer size decreased?	**YES**	**NO**
Is there formation of a new ulcer?	**YES**	**NO**
Has the ulcer completely healed?	**YES**	**NO**
Is there a decrease in glycaemic control?	**YES**	**NO**
Is there a decrease in renal function?	**YES**	**NO**
Has the patient commenced dialysis	**YES**	**NO**
Is there increased AGE formation present?	**YES**	**NO**
Are there differences in the ROM of joints?	**YES**	**NO**
Is there deterioration of vascular function?	**YES**	**NO**
Is there a deterioration of neurological function?	**YES**	**NO**
Has there been a reduction in treatment frequency?	**YES**	**NO**
Has there been an increase in treatment frequency?	**YES**	**NO**
Was a biopsy conducted?	**YES**	**NO**
Is there infection present?	**YES**	**NO**
Has haematological status deteriorated critically? I.e.- Lipid profile, WCC, CRP, ESR	**YES**	**NO**
Is the participant still suitable for the study?	**YES**	**NO**
Next visit date provided	______/______/______/	

If the participant is no longer suitable for the study, please send a letter of thanks for being involved in the study, otherwise provide date and time of next consultation.

Next consultation visit-

End of Study:

Did the participant complete all aspects of the study?

Diabetes and Foot ulceration Study		
Unique Identification Number:	UR:	Name:
DOB:	Initials:	

☐ Yes	☐ No
☐ According to the protocol	☐ Participant withdrew*
☐ With protocol deviations or violations (ensure these are recorded on the protocol deviation page)	☐ Lost to follow-up
	☐ Participant deceased (ensure an SAE has been recorded & reported)
	Patient declined further involvement*
	☐ Other*
*Comment:	

Has a letter of thanks been sent to the participant along with a 1-page summary of significant findings?

YES

NO

Has a letter of thanks been sent to the participant along with a 1-page summary of significant findings to the referring practitioner?

YES

NO

Diabetes and Foot Ulceration Study		
Unique Identification Number:	UR:	Name:
DOB:	Initials:	

Protocol Deviations:

Deviation Number	CRF Page Number	Deviation	Recorded by
1			
2			
3			
4			
5			
6			
7			
8			
9			
10			
11			
12			
13			
14			
15			
16			
17			
18			
19			
20			

Diabetes and Foot Ulceration Study		
Unique Identification Number:	UR:	Name:
DOB:	Initials:	

End of CRF:

Data collection completed by (Name)	
Signature	
Date completed	□□/□□□/□□□□
Principle Investigator (Name):	
Signature	
Date	□□/□□□/□□□□
